# Fucose as a potential therapeutic molecule against the immune-mediated inflammation in IgA nepharopathy: An unrevealed link

**DOI:** 10.3389/fimmu.2022.929138

**Published:** 2022-08-17

**Authors:** Jianbo Qing, Xueli Hu, Changqun Li, Wenzhu Song, Hasna Tirichen, Hasnaa Yaigoub, Yafeng Li

**Affiliations:** ^1^ The Fifth Clinical Medical College of Shanxi Medical University, Taiyuan, China; ^2^ Department of Nephrology, Shanxi Provincial People’s Hospital (Fifth Hospital) of Shanxi Medical University, Taiyuan, China; ^3^ School of Public Health, Shanxi Medical University, Taiyuan, China; ^4^ Institutes of Biomedical Sciences, Shanxi University, Taiyuan, China; ^5^ Core Laboratory , Shanxi Provincial People’s Hospital (Fifth Hospital) of Shanxi Medical University, Taiyuan, China; ^6^ Shanxi Provincial Key Laboratory of Kidney Disease, Taiyuan, China; ^7^ Academy of Microbial Ecology, Shanxi Medical University, Taiyuan, China

**Keywords:** IgA nephropathy, immune-mediated inflammation, hub genes, machine learning, fucose

## Abstract

**Background:**

IgA nephropathy (IgAN) is an autoimmune disease that affects people of any age and is an important cause of end-stage renal disease. However, the pathogenesis and pathophysiology of IgAN is not clear. This article aimed to explore the immune-mediated inflammation and genetic mechanisms in IgAN.

**Methods:**

The transcriptome sequencing data of IgAN glomeruli in the Gene Expression Omnibus database were downloaded. Single-sample gene set enrichment analysis was used to estimate the immune microenvironment of the merged microarray data and GSE141295. IgAN samples were divided into two clusters by cluster analysis. “limma” and “DEseq2” package in R were used to identify differentially expressed genes (DEGs). The weighted gene co-expression network analysis (WGCNA) was used to identify the co-expression modules related to inflammation in IgAN. R software package “clusterProfiler” was used for enrichment analysis, whereas Short Time-Series Expression Miner (STEM) analysis was used to identify the trend of gene expression. Machine-learn (ML) was performed using the shiny app. Finally, Drug Signatures Database (DSigDB) was used to identify potential molecules for treating IgAN.

**Results:**

The infiltration of macrophages in IgAN glomeruli was increased, whereas CD4+ T cells, especially inducedregulatory T cells (iTregs) were decreased. A total of 1,104 common DEGs were identified from the merged data and GSE141295. Brown module was identified to have the highest inflammatory correlation with IgAN using WGCNA, and 15 hub genes were screened from this module. Among these 15 hub genes, 14 increased with the severity of IgAN inflammation based on STEM analysis. Neural network (nnet) is considered as the best model to predict the severity of IgAN. Fucose identified from DSigDB has a potential biological activity to treat IgAN.

**Conclusion:**

The increase of macrophages and the decrease of iTregs in glomeruli represent the immune-mediated inflammation of IgAN, and fucose may be a potential therapeutic molecule against IgAN because it affects genes involved in the severe inflammation of IgAN.

## Introduction

IgA nephropathy (IgAN) has become a considerable public health issue worldwide as it affects people of any age. Approximately 30% of patients with IgAN develop end-stage renal disease (ESRD) within 20 years of diagnosis (1). As the most prevalent glomerulonephritis worldwide, genetic factors, mucosal immune responses, infections, and gut microbiome participate in its occurrence and development (2, 3). Although the precise pathogenetic mechanisms are not sufficiently clear (4), undoubtedly, immunological factors play a critical role in all stages of IgAN development (5). Poorly O-galactosylated IgA1, produced by abnormal mucosal immunity, will deposit in the glomerular mesangium, leading to the activation of various immune cells and the release of inflammatory mediators in the kidney (6). Subsequently, glomerular and tubule function will be impaired (7).

Immune-mediated inflammation greatly affects the pathophysiology of IgAN. However, limited understanding poses great challenges for effective treatment of IgAN (8). Currently, there are not approved therapies yet, both optimized supportive care and immunosuppressive therapy have many deficiencies (9). It is crucial to explore the immune microenvironment and potential markers of inflammation in IgAN to clarify the pathogenesis of IgAN and seek effective treatment such as targeted therapy (10).

In this study, we collected all transcriptome sequencing data of IgAN glomeruli from the Gene Expression Omnibus (GEO) database (11). In addition, bioinformatics such as single-sample gene set enrichment analysis (ssGSEA), co-expression analysis, and machine learning (ML) were used to explore the immune characteristics of IgAN glomeruli and screen out the signature genes that may represent inflammation in IgAN glomeruli.

## Methods

### Data collection and processing

The keyword “IgA Nephropathy” was used to search IgAN gene expression profiles in the GEO database. For accuracy and comprehensiveness, all transcriptome sequencing data of IgAN glomeruli were obtained from the GEO database. Five datasets [including four microarray datasets and one RNA sequencing (RNA-seq) dataset] were downloaded (**Table 1**). Then, gene symbols conversion and log2 transformation were performed for gene expression profiling. In addition, R software package “inSilicoMerging” was used for the combination of multiple datasets. Then, the “combat” function was used to remove the batch effect to finally get “data1” (**Supplementary Table 1**) (12). Although RNA-seq and microarray expression levels may appear different, transforming them into biologically relevant gene set enrichment scores significantly increases their correlation (13). Thus, two types of sequencing data were combined to acquire more reliable results.

**Table 1:** The information of five datasets obtained from GEO database.

Datasets	Platform	Samples	Tissue	Type
GSE93798	GPL22945	20 patients with IgAN and 22 controls	Glomeruli	Microarray
GSE37463	GPL14663	27 patients with IgAN and 27 controls	Glomeruli	Microarray
GSE99340	GPL19109	26 patients with IgAN	Glomeruli	Microarray
GSE104948	GPL24120	27 patients with IgAN and 20 controls	Glomeruli	Microarray
	GPL22945		Glomeruli	
GSE141295	GPL16791	14 patients with IgAN and 10 controls	Glomeruli	RNA-seq

### Single-sample gene set enrichment analysis

As CIBERSORT and other methods could not yield enough results of T cell subtypes, ImmuCellAI (Immune Cell Abundance Identifier) was used to perform ssGSEA on the combined microarray data and RNA-seq data, which is a tool to estimate the abundance of 24 immune cells from gene expression datasets. The 24 immune cells are composed of 18 T-cell subtypes and six other immune cells. The marker gene sets for immune cell types were obtained from Miao et al. (**Supplementary Table 2**) (14).

### Immune cell clustering of IgAN

Although the GEO database does not have sufficient clinical information of these samples, cluster analysis can help find subclasses of disease. Thus, “ConsensusClusterPlus” was used to perform cluster analysis, which is a class discovery tool with confidence assessments and item tracking, using agglomerative pam clustering with a one–Pearson correlation distances and resampling 80% of the samples for 10 repetitions (15). The optimal number of clusters was determined using the empirical cumulative distribution function plot. Comparison of identified clusters of immune cell differences to distinguish their immune characteristics was conducted.

### Differential expression analysis for IgAN

“limma” in R software was used to identify the differentially expressed genes (DEGs) between IgAN and controls in data1 (16). “DESeq2 (version 1.32.0)” package was used to conduct difference analysis in GSE141295 (17). Genes with a criterion of *P <* 0.05 were considered as DEGs. Then, the results of the two parts were combined to get both upregulated and downregulated common DEGs in two types of sequencing data. We named the expression matrix of these genes in IgAN samples of data1 as “data2” (**Supplementary Table 3**).

### Co-expression analysis between DEGs and IgAN clusters

Data2 was used to construct a scale-free co-expression network through weighted gene co-expression network analysis (WGCNA) using the R package “WGCAN” (18). First, the Pearson’s correlation matrices and average linkage method were both performed for all pair-wise genes. Then, a weighted adjacency matrix was constructed using a power function A_mn=|C_mn|^β (C_mn = Pearson’s correlation between Gene_m and Gene_n; A_mn= adjacency between Gene m and Gene n). β was a soft-thresholding parameter that could emphasize strong correlations between genes and penalize weak correlations. After choosing the power of 4, the adjacency was transformed into a topological overlap matrix (TOM), which could measure the network connectivity of a gene defined as the sum of its adjacency with all other genes for network generation, and the corresponding dissimilarity (1-TOM) was calculated. To classify genes with similar expression profiles into gene modules, average linkage hierarchical clustering was conducted according to the TOM-based dissimilarity measure with a minimum size (gene group) of 25 for the genes dendrogram. To further analyze the module, we calculated the dissimilarity of module eigen genes, chose a cut line for module dendrogram, and merged some modules. Other parameters include R square-cut = 0.85 and deep split = 2. Finally, we identified the module with the highest correlation coefficient and screened the hub genes (19).

### Enrichment analysis

R software package “clusterProfiler” (version 3.14.3) was used for enrichment analysis to obtain the results of Kyoto Encyclopedia of Genes and Genomes (KEGG) and Gene Ontology (GO) analysis (20). Set the minimum gene set to 5 and the maximum gene set to 5,000. *P*-value < 0.05 and a false discovery rate (FDR) < 0.25 were considered statistically significant.

### Short time-series expression miner analysis

Short Time-Series Expression Miner (STEM) analysis was performed to cluster the hub genes with the same time trend in clusters and controls in data1; P < 0.05 was considered statistically significant clustering (21). The significantly clustered genes showed a gradual upregulation or downregulation trend.

### Construction and assessment of machine learning models

For better predicting and evaluating the inflammation in IgAN, “Machine-learn” in the shiny app (https://shiny.hiplot.com.cn/mach-learn/) was used to construct six ML models (SvmLinear, SvmPoly, Neural Network, RandomForest, K-NN, and Naive Bayes) based on hub genes. Cross-validation (CV) folds were set to 10 (22). Finally, the best model was identified according to accuracy and kappa-value.

### Identification of potential molecules for IgAN

The inflammatory characteristics of IgAN were represented by hub genes identified by WGCNA. The Drug Signatures Database (DSigDB) in the Enrichr (https://amp.pharm.mssm.edu/Enrichr/) platform was used to screen the molecules, which could regulate the expression of hub genes and identify candidate molecules for IgAN treatment (23).

## Results

### Data collection and processing

**Figure 1:**
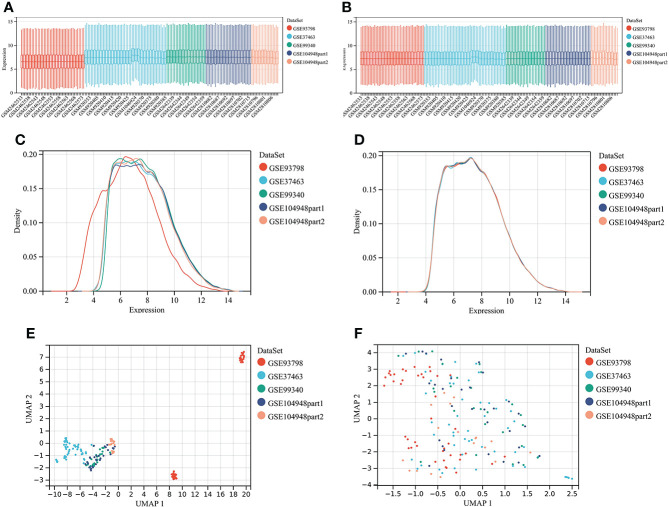
Merging datasets and removing batch effect. **(A)** The box plot of sample distribution of each datasets before the removal of batch effect, which indicated the differences of sample distribution and the existence of batch effect. **(B)** The box plot of sample distribution of each datasets after the removal of batch effect, which indicated that data distribution tends to be consistent across datasets, with the median in a line. **(C)** The density plot of sample distribution of each datasets before the removal of batch effect, which indicated the differences of sample distribution and the existence of batch effect. **(D)** The density plot of sample distribution of each datasets after the removal of batch effect, which indicated that data distribution tends to be consistent across datasets, with the close mean and variance. **(E)** The UAMP of sample distribution of each datasets before the removal of batch effect; samples from individual datasets are clustered separately, which indicated the existence of batch effect. **(F)** The UAMP of sample distribution of each datasets after the removal of batch effect; the samples of each datasets were clustered together, suggesting a good removal of batch effect.

Four microarray datasets were merged to a new matrix “data1” using package “inSilicoMerging”, which consisted of 100 patients with IgAN and 70 healthy controls. Box plot, density plot, and uniform manifold approximation and projection (UMAP) plot were generated to show differences between samples before and after batch effect removal (**Figure 1**). It can be observed that the sample distribution of each dataset differs greatly before the removal of the batch effect, suggesting the existence of the batch effect. After the removal of the batch effect, the data distribution of each dataset tends to be consistent.

### Single-sample gene set enrichment analysis

The abundance of 24 types of immune cells in data1 and GSE141295 was calculated using ImmuCellAI, including 10 kinds of layer1 cells (DC, B cell, monocyte, macrophage, NK cell, neutrophil, CD4+ T cell, CD8+ T cell, NKT, and Tgd) and 14 kinds of layer2 cells, which contained 14 T cell subtypes (CD naive T cell, Tr1, nTreg, iTreg, Th1, Th2, Th17, Tfh, CD8 naive T cell, Tc, Tex, MAIT, Tcm, and Tem).

**Figure 2:**
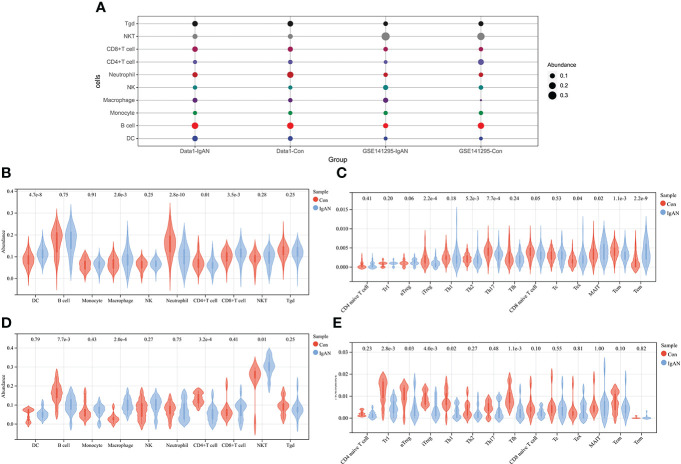
ssGSEA of data1 and GSE141295. **(A)** Differences in 10 layer1 immune cell infiltration between IgAN and controls. Each row represents a type of immune cell; each column, a different group. **(B)** Differences in abundance of 10 types of layer1 immune cells between IgAN and controls of data1. **(C)** Differences in abundance of 14 types of layer2 immune cells between IgAN and controls of data1. **(D)** Differences in abundance of 10 types of layer1 immune cells between IgAN and controls of GSE141295. **(E)** Differences in abundance of 14 types of layer2 immune cells between IgAN and controls of GSE141295.

**Figure 2A** demonstrated the abundance of 10 layer1 immune cells in data1 and GSE141295. The DC, macrophage, CD8+ T cell, Tex, MAIT, and Tem were increased in IgAN samples of data1, whereas the CD4+ T cell, neutrophils, iTreg, nTreg, Tcm, Th2, and Th17 were reduced (**Figures 2B, C**). Meanwhile, macrophage and NKT were increased, and B cell, CD4+ T cell, Tr1, nTreg, iTreg, Th1, and Tfh were reduced in IgAN samples of GSE141295 (**Figures 2D, E**). It was suggested that the immune signatures of IgAN may be represented by the increase in macrophages and a decrease in CD4+ T cells, particularly iTreg cells based on the ssGSEA results of data1 and GSE141295.

### Immunoinfiltration clustering of IgAN

**Figure 3:**
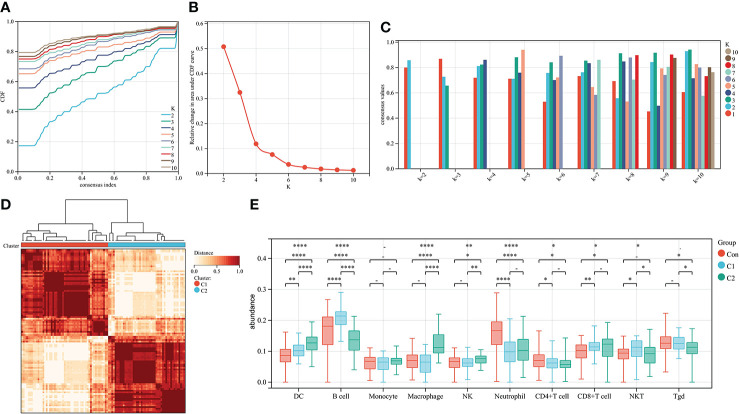
Cluster analysis of 100 IgAN samples in data1 based on the abundance of immune cells. **(A)** The CDF curve of different K-values; the CDF delta has the slowest downward trend when K-value = 2. **(B)** The relative change in area under CDF curve; as the K-value increased, the area under the CDF curve increases gradually. **(C)** The bar graph of the consistency within the clusters; the number of clusters with the highest average consistency within the group was K-value = 2. **(D)** The heat map of clusters; there were 53 IgAN samples in cluster 1, whereas there were 47 IgAN samples in cluster 2. **(E)** The differences in abundance of 10 types of layer1 immune cells between controls, cluster 1, and cluster 2. *P ≤ 0.05, **P ≤ 0.01, ****P ≤ 0.0001.

To further explore the genetic mechanisms of IgAN, cluster analysis was performed on 100 IgAN samples in data1 based on the abundance of 24 types of immune cells obtained from ssGSEA. In the CDF curve of different K-values, the CDF delta has the slowest downward trend when K-value = 2 (**Figure 3A**), and the area under the CDF curve increased with the increase of the K-value (**Figure 3B**). We need to keep the CDF delta decline as slow as possible on the premise of keeping the area as large as possible. Ultimately, the number of clusters was selected as K = 2 for achieving the highest average consistency within the groups (**Figure 3C**). One-hundred IgAN samples were divided into two clusters (**Figure 3D**), 53 samples in cluster 1, and 47 samples in cluster 2 (**Supplementary Table 4**).

We then compared the abundance of immune cells in cluster 1 and cluster 2. DCs, macrophages, and NK cells in cluster 2 were significantly higher than those in cluster 1. There was no difference in macrophages and NK cells of cluster 2 compared with the controls (**Figure 3E**). Thus, cluster 2 is considered to represent severe inflammation, whereas cluster 1 represented mild inflammation in IgAN samples.

### Differential expression analysis for IgAN

**Figure 4:**
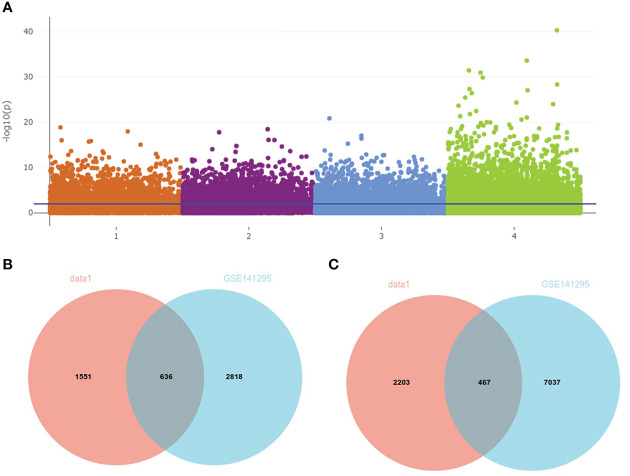
Identification of common DEGs in data1 and GSE141295. **(A)** The Manhattan plot of DEGs in data1 and GSE141295. 1: 1,553 upregulated genes in the IgAN samples of data1; 2: 2,203 downregulated genes in the IgAN samples of data1; 3: 2,818 upregulated in the IgAN samples of GSE141295; 4: 7,097 downregulated in the IgAN samples of GSE141295. **(B)** The Venn diagram of 636 common upregulated genes in data1 and GSE141295. **(C)** The Venn diagram of 467 common downregulated genes in data1 and GSE141295.

A total of 3,754 DEGs between IgAN and the control group in data1 were identified with *P <* 0.05, including 1,553 upregulated and 2,203 downregulated genes. In addition, 2,818 upregulated and 7,097 downregulated genes were obtained from GSE141295 (**Figure 4A**); DEGs of data1 and GSE141295 were combined to screen 1,104 common DEGs, which consisted of 637 upregulated and 467 downregulated genes (**Figures 4B, C** **)**. Then, the expression matrix of 1,104 common DEGs from 100 IgAN samples in data1 was selected to generate data2 for further analysis.

### Co-expression analysis between DEGs and IgAN clusters

**Figure 5:**
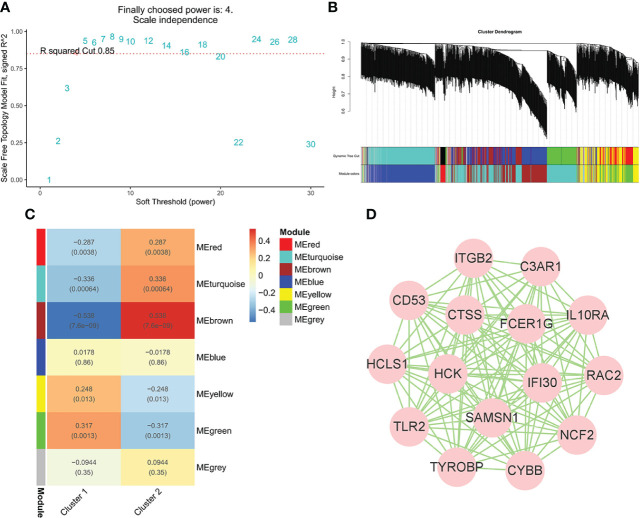
WGCNA of IgAN clusters in data. **(A)** Identification of soft threshold, β = 4 was considered the most suitable. **(B)** The cluster dendrogram of co-expression genes in clusters of 100 IgAN samples. **(C)** Module–trait relationships in clusters of 100 IgAN samples. Each cell contains corresponding correlation and p-value. **(D)** The network of 15 hub genes in the brown module based on MM = 0.9.

To identify the genes significantly associated with IgAN clusters, 1,104 genes in data2 were divided into seven modules using WGCNA based on β = 4 (**Figures 5A, B**). In addition, the brown module was positively correlated with cluster 2 and negatively correlated with cluster 1, which had the highest correlation with igAN cluster and contained 219 genes (**Figure 5C**). Furthermore, we calculated the correlation between module feature vectors and gene expression to obtain hub genes, based on the cutoff criteria (|MM| > 0.9). Fifteen genes with high connectivity in the clinical significant module were identified as hub genes, which were considered to be associated with severe inflammation in IgAN. Cytoscape 3.8.2 was used to show the correlations between them (**Figure 5D**).

### Enrichment analysis

**Figure 6:**
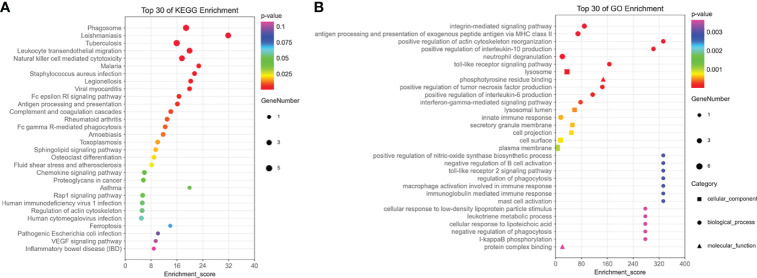
The enrichment analysis of 15 hub genes in the brown module. **(A)** The KEGG enrichment analysis of 15 hub genes in the brown module. **(B)** The GO enrichment analysis of 15 hub genes in the brown module, including cellular component, biological process, and molecular function.

The results of enrichment analysis indicated that 15 hub genes were related to immune processes such as NK cell–mediated cytotoxicity, complement and coagulation cascades, and cell killing pathways in KEGG (**Figure 6A**). In terms of GO, 15 hub genes were involved in, interleukin-6 (IL-6), IL-10, Toll-like receptor signaling pathway, interferon-gamma–mediated signaling pathway, and macrophage activation involved in immune response production (**Figure 6B**).

### Short time-series expression miner analysis

**Figure 7:**
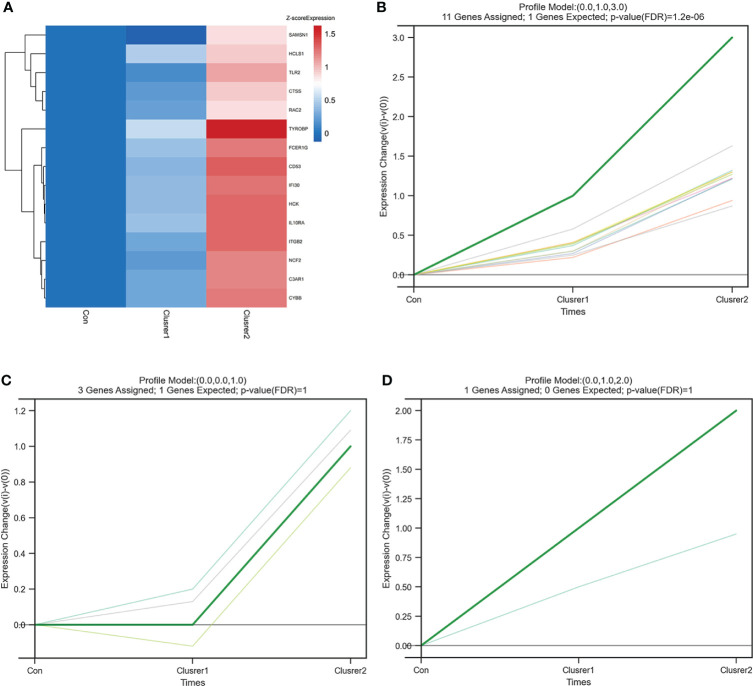
STEM analysis of 15 hub genes. **(A)** The heat map of 15 hub gene expression trends. In addition to SAMSN1, the expression of the other 14 hub genes increased gradually from control group to cluster 1 and then to cluster 2. **(B)** The line chart of six gene expressions in trend 1, including C3AR1, CD53, CTSS, CYBB, FCER1G, HCK, IFI30, IL10RA, ITGB2, RAC2, and TYROBP. **(C)** The line chart of six gene expressions in trend 2, including NCF2, SAMSN1, and TLR2. **(D)** The line chart of six gene expressions in trend 3, including HCLS1.

STEM analysis was used to determine the alteration in 15 hub genes during the progression from healthy controls to different IgAN clusters (**Supplementary Table 5**). Among them, C3AR1, CYBB, CTSS, IGTB2, FCER1G, TYROBP, CD53, RAC2, HCK, IFI30, IL10RA, NCF2, TLR2, and HCLS1 showed a trend of gradual increase with different IgAN clusters (**Figure 7A**). C3AR1, CD53, CTSS, CYBB, FCER1G, HCK, IFI30, IL10RA, ITGB2, RAC2, and TYROBP were in trend 1, NCF2, SAMSN1, and TLR2 were in trend 2, whereas HCLS1 was in trend 3 (**Figures 7B–D**). The results of STEM analysis suggested that these 14 genes may be associated with different immune infiltration of IgAN alterations. These genes may be potential inflammation biomarkers in IgAN.

### Construction and assessment of machine learning models

**Figure 8:**
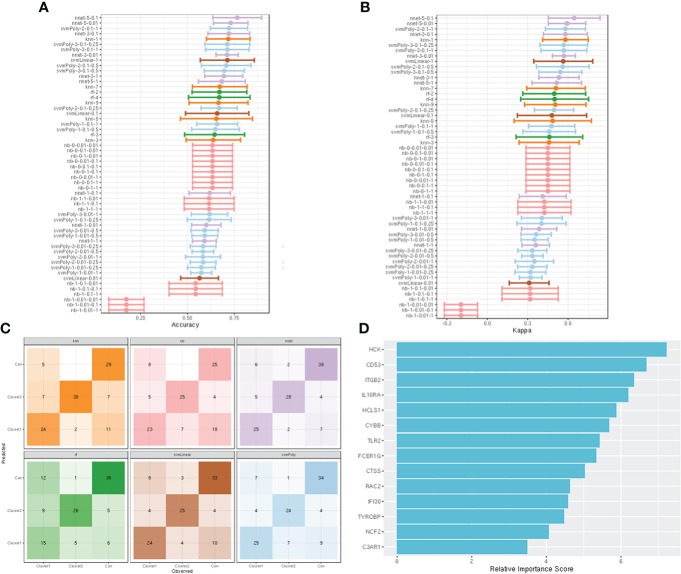
construction and evaluation of ML models. **(A)** The cross-validation results of the accuracy of all models. **(B)** The cross-validation results of the kappa-value of all models. **(C)** The observed *vs*. predicted (best candidate for algorithm) of all models. **(D)** Feature importance of genes in the ML models.

Fourteen hub genes that increased with different IgAN clusters were selected to construct ML models. The model rank and prediction–observation values of six models were generated on the basis of CV = 10 (**Figures 8A, B**). The value of observed *vs*. predicted is shown in **Figure 8C**, and **Figure 8D** shows the feature importance. Considering accuracy and kappa-values (**Table 2**), Nnet-5-0.1 was the best model for predicting the inflammation in IgAN based on 14 hub genes, which were significantly correlated with the severity of inflammatory infiltration in IgAN glomeruli. This model may be of great value in predicting the severity of IgAN glomeruli inflammation in the future.

**Table 2:** The accuracy and kappa-value of top 10 models.

Model	Accuracy	Kappa	AccuracySD	KappaSD
nnet-5-0.1	0.766	0.645	0.13	0.192
nnet-5-0.01	0.731	0.592	0.092	0.139
svmPoly-2-0.1-1	0.722	0.58	0.099	0.148
nnet-3-0.1	0.721	0.58	0.111	0.163
knn-1	0.718	0.58	0.116	0.169
svmPoly-3-0.1-0.25	0.713	0.568	0.120	0.176
svmPoly-3-0.1-1	0.713	0.569	0.116	0.170
nnet-3-0.01	0.712	0.569	0.059	0.085
svmLinear-1	0.713	0.564	0.144	0.219
svmPoly-2-0.1-0.5	0.707	0.560	0.132	0.195

### Identification of potential molecules for IgAN

Enrichr platform is used to identify potential molecules for IgAN using hub genes. The molecules that could regulate the expression of hub genes were collected from DSigDB (**Supplementary Table 6**). **Table 3** shows the top 10 potential molecules from DSigDB; among them, fucose was considered as a candidate molecule for the treatment of IgAN as it has the highest odds ratio and the highest combined score.

**Table 3:** Suggested top 10 small molecules for IgAN.

Term	P-value	Odds Ratio	Combined Score
fucose TTD 00008125	6.92E-05	208.0208333	1992.61613
pergolide HL60 UP	6.57E-10	64.52786885	1364.342173
GLYOXAL CTD 00006046	0.007674931	153.6615385	748.3003459
Eckol CTD 00002503	0.007674931	153.6615385	748.3003459
ADAPALENE CTD 00002588	0.009064489	128.0384615	602.2149226
methylglyoxal CTD 00006677	0.009758591	118.183432	547.142878
astemizole HL60 UP	5.69E-04	67.81292517	506.6875974
TRIMELLITIC ANHYDRIDE CTD 00000808	0.010452241	109.7362637	500.5003914
PCB 118 CTD 00002737	0.010452241	109.7362637	500.5003914
alfacalcidol CTD 00000387	0.010452241	109.7362637	500.5003914

## Discussion

IgAN is an autoimmune disease that is driven by environment, genetics, and immunity, particularly the dysregulations in the adaptive and innate immune systems  (5). Although it has been discovered and studied for many years, there are still limitations on its pathogenesis, pathophysiology, and treatment (24). Exploring the immune-mediated inflammation in IgAN glomeruli would bring new insights and breakthroughs to the protecting of glomerular function and personalized treatment.

To improve the accuracy and persuasiveness of the study, all IgAN glomeruli-related microarray data in the GEO database were downloaded and combined into new data in addition to a RNA-seq dataset. There were differences in ssGSEA results between microarray data and RNA-seq data, but both of them lead to the same conclusion that is macrophage infiltration increased while CD4+ T cell infiltration decreased in the IgAN glomeruli.

Macrophages can be used as an important indicator to estimate the development and prognosis of renal diseases due to their involvement in the development of renal fibrosis (25), which is a necessary process for all chronic kidney disease to develop into ESRD (26). Stimulated by inflammatory signals, circulating monocytes migrate and infiltrate into the kidney and then differentiate into macrophages (27). Macrophages could differentiate into M0, M1, and M2 types (28). Immune complexes and necrotic cells can activate innate immune receptors and promote the differentiation of M1 macrophages, which promotes a series of inflammatory responses and leads to kidney damage by secreting proinflammatory cytokines such as TNF-α, IL-1, IL-6, IL-12, and chemokines (29). As the development of inflammation and the production of anti-inflammatory factors, the macrophages polarize toward M2 macrophages, which limit inflammatory response, promote renal repair, and cause fibrosis (30). Macrophages play a critical role in glomerular inflammation and glomerulosclerosis, and anti-apoptosis inhibitor of macrophage therapy could inhibit the progression of kidney inflammation and renal fibrosis (31). This reveals the importance of macrophages in immune-mediated inflammation in IgAN. The abundance of macrophages represents the severity of renal inflammation in IgAN, which provides theoretical support for our subsequent cluster analysis and targeted macrophage therapy.

In contrast to the increase of macrophages, the abundance of CD4+ T cells was significantly reduced compared with the controls in IgAN glomeruli. CD4+ T cells participate in the IgAN through differentiating into helper T cells (Th) and regulatory T cells (Treg) (32). Notably, as an important subtype of CD+ T cells, iTreg was significantly reduced in the glomeruli of IgAN. T cells are induced to differentiate *in vitro* to produce iTregs that exert immunosuppressive actions through secreting cytokines, such as IL-4, IL-10, and TGF-β, and play an important role in immune homeostasis and induction of immune tolerance (33). A number of research revealed that the decrease of Tregs may be involved in the occurrence of IgAN (34, 35). Some studies indicated not only a deficient quantity but also poor immunosuppressive function of Tregs in IgAN (36). In addition, The decrease of Tregs always signifies an increased abundance of Ths. Among the various Ths, Th17 deserves the most attention, as the balance of Th17 and Treg *in vivo* maintains immune homeostasis and they can be transformed into each other under certain conditions (37, 38). The dual defects in the quantity and function of Tregs make the inflammation more active in the kidney, which could be an important cause of IgAN progression (36). Restoring immune homeostasis and tolerance through the amplification and induction of Tregs may be an important way to cure or control IgAN (39). It is worth mentioning that the inflammation in IgAN glomeruli is mediated by a combination of immune cells, but perhaps the most dazzling are macrophages and T cells. Changes in macrophages and T cells may be the most valuable and effective therapeutic targets.

On the basis of the results of ssGSEA, cluster analysis was performed on IgAN samples in data1 and obtained two clusters. Macrophages and NK cells in cluster 2 were significantly higher than those in cluster 1. Cluster 1 was defined as mild inflammation and cluster 2 as severe inflammation. In addition, 637 upregulated and 467 downregulated genes were identified from data1 and GSE141295. The above results were then used for WGCNA to explore the gene modules closely related to glomerular inflammation.

The brown module had the highest correlation with IgAN clusters, and 15 hub genes were screened from it according to appropriate thresholds, which were considered to be markers of severe inflammation in IgAN glomeruli. KEGG enrichment analysis indicated that these 15 hub genes were involved in various immune processes such as NK cell–mediated cytotoxicity and cell killing pathways, which are related to the progression and prognosis of IgAN (40). In addition, hub genes were involved in the complement and coagulation cascades, which play a crucial role in IgAN progression and renal function decline (41). In terms of GO enrichment analysis, 15 hub genes were associated with the production of IL-6 and IL-10, macrophage activation involved in immune response, and Toll-like receptor signaling pathway, which are important components of innate and adaptive immunity and critical for the pathogenesis of IgAN (42). It is worth mentioning that IL-6 involves in the etiopathogenesis of IgAN through the production of Gd-IgA1 and regulation of mesangial cell proliferation (43), whereas IL-10 represses proinflammatory responses and limits kidney disruptions caused by inflammation (44). Although almost all lymphocytes can synthesize IL-10, the most important sources *in vivo* are activated mononuclear macrophages and CD4+ T cells (45). These results indicated that the hub genes are closely related to the changes in the abundance and activation of CD4+ T cells and macrophages in the immune microenvironment of IgAN, which could be used as potential therapeutic targets.

Searching for reliable biomarkers is the basis of diagnosis and targeted therapy for IgAN. We identified 14 genes from 15 hub genes using STEM analysis, which was beneficial for predicting and evaluating the inflammation in IgAN glomeruli. ML has been widely used in medicine to construct diagnostic and predictive models of diseases (46). After testing and evaluating six models, neural network (nnet) was considered as the most valuable for the prediction of inflammation in IgAN glomeruli due to its highest accuracy and kappa-values. It is widely used for building prediction models from microarray data (47). The research on the immune microenvironment and the prediction model is expected to promote the exploration of the pathogenesis and personalized treatment of IgAN.

Angiotensin-converting enzyme inhibitor or angiotensin II receptor blocker benefits in controlling blood pressure and reducing proteinuria. However, they cannot improve the inflammation of the kidney. Although immunosuppressants and monoclonal antibodies have been used to inhibit the inflammatory response and progression of IgAN, there is debate about their safety and effectiveness (10). Thus, it is critical to explore effective drugs for improving the symptoms and prognosis of patients with IgAN.

Fucose in DSigDB was identified as a potential molecule for treating IgAN based on 14 hub genes. Fucose is a monosaccharide present abundantly in gut glycoproteins and mediates the host–microbe symbiosis that could suppress the virulence of pathogens and pathobionts and improves both gut-centered and systemic infection and inflammation (48). Furthermore, fucose plays an important role in immunoregulation of renal disease by reducing the deposition of complement C3 on renal tubules and infiltration of immune cells (49), which is beneficial to the therapeutic intervention of IgAN (50). Moreover, the activation of macrophage can be inhibited by fucose, and it is an important regulator of intestinal mucosal immunity (51). Fucose plays a critical role in mucosal immunity through its immunocompetence of anti-infection and anti-inflammation (52), which is of great significance to prevent the occurrence and progression of IgAN from the pathogenesis. In addition, fucose is also a powerful antioxidant that reduces tissue damage caused by inflammation and protects renal function (53). The excellent biological activity of fucose may become an effective treatment for IgAN in the future.

There are also certain limitations in our study; the data obtained from GEO lack adequate clinical information, which makes more detailed grouping and exploration difficult. Further molecular experiments for the validation of genes and molecules are missing, and large clinical samples will be collected to verify our findings in the future. In addition, fucose as a potential therapeutic molecule for IgAN is predicted based on the DSigDB and lacks experimental verification, and we plan to conduct subsequent experiments in animal models.

In conclusion, the present study demonstrated that the increase of macrophages and decrease of iTregs are immune signatures in IgAN glomeruli. We identified 15 hub genes associated with severe inflammation and used 14 of them to construct a nnet model to predict and evaluate the inflammation in IgAN. Finally, we predict a molecule called fucose that may become an effective drug for IgAN in the future.

## Data availability statement

The datasets presented in this study can be found in online repositories. The names of the repository/repositories and accession number(s) can be found in the article/**Supplementary Material**.

## Author contributions

JQ processed the data and drafted the paper. CL and XH helped filter datasets for the manuscript. WS, HT, and HY helped polish the manuscript. YL was responsible for final review. All authors contributed to the article and approved the submitted version.

## Funding

This work was supported by the National Natural Science Foundation of China (82170716) and the Research Project Supported by Shanxi Scholarship Council of China(2020-183).

## Acknowledgments

We express our sincere gratitude to those who helped us a lot during our writing process.

## Conflict of interest

The authors declare that the research was conducted in the absence of any commercial or financial relationships that could be construed as a potential conflict of interest.

## Publisher’s note

All claims expressed in this article are solely those of the authors and do not necessarily represent those of their affiliated organizations, or those of the publisher, the editors and the reviewers. Any product that may be evaluated in this article, or claim that may be made by its manufacturer, is not guaranteed or endorsed by the publisher.
